# Importance of Determining Vascular Endothelial Growth Factor Serum Levels in Children with Infantile Hemangioma

**DOI:** 10.3390/medicina59111914

**Published:** 2023-10-29

**Authors:** Arnes Rešić, Nikolina Benco Kordić, Jasna Obuljen, Marko Bašković

**Affiliations:** 1 Department of Pediatrics, Children’s Hospital Zagreb, Ulica Vjekoslava Klaića 16, 10 000 Zagreb, Croatia; 2 University Department of Health Studies, University of Split, Ruđera Boškovića 35, 21 000 Split, Croatia; 3 Department of Medical Biochemistry and Hematology, Children’s Hospital Zagreb, Ulica Vjekoslava Klaića 16, 10 000 Zagreb, Croatia; 4 Department of Pediatric Surgery, Children’s Hospital Zagreb, Ulica Vjekoslava Klaića 16, 10 000 Zagreb, Croatia; 5 Scientific Centre of Excellence for Reproductive and Regenerative Medicine, School of Medicine, University of Zagreb, Šalata 3, 10 000 Zagreb, Croatia; 6 School of Medicine, University of Zagreb, Šalata 3, 10 000 Zagreb, Croatia

**Keywords:** vascular endothelial growth factor, infantile hemangiomas, angiogenesis, children, proliferation phase, involution phase

## Abstract

*Background and Objectives*: A potential role of vascular endothelial growth factor (VEGF) in the pathophysiology of infantile hemangiomas (IH) is thought to be plausible. The primary objective of this study was to investigate the importance of determining VEGF serum levels at various stages of IH growth in children. *Materials and Methods*: A nested case–control study was conducted. For the purposes of the researched target group, samples of fifty (N = 50) children with IH without associated diseases at different stages of hemangioma growth (proliferative and involutional stages) were used. The control group consisted of one hundred (N = 100) healthy children comparable in terms of age and sex, in whom the existence of IH and vascular malformations was ruled out via clinical examination. An immunoassay (ELISA) was used to determine VEGF serum levels in hemangioma growth’s proliferation and involution phases. *Results*: A comparison of serum levels of VEGF in the phases of proliferation and involution in the group of patients with IH did not show a statistically significant difference (*p* = 0.171). The control group had significantly higher serum VEGF levels than the patient group in both the proliferation phase (*p* = 0.009) and the involution phase (*p* = 0.019). In the proliferation phase, a multivariate regression model explained 15% of the variance in the dependent variable, without significant predictor variables, while in the involution phase, it explained 21% of the variance in the dependent variable, and the history of invasive prenatal procedures stood out as a significant predictor variable positively associated with serum VEGF levels (beta coefficient = 0.33; *p* = 0.043). *Conclusions*: Although IH is thought to be the result of the dysregulation of angiogenesis and vasculogenesis under the influence of angiogenic factors, especially VEGF, this study did not demonstrate that VEGF serum levels in the proliferation phase of hemangioma growth were higher than those in the involution phase, or in relation to the control group.

## 1. Introduction

Infantile hemangiomas (IH) are the most common benign vascular tumors in children, with an incidence of up to 10% [1]. They are characterized by rapid growth in infancy (proliferation) and the stopping of growth after the first year of life or later in childhood (involution). The pathogenesis of IH is still unknown. Immunohistochemical studies confirm that IHs are of vascular endothelial origin. Histologic analyses indicate that they are highly cellular tumors, composed of disorganized blood vessels and immature endothelial cells that are, in terms of morphology and protein expression, similar to fetal endothelial cells with the possibility of differentiation [2]. IHs are also composed of non-endothelial cells, stromal cells exhibiting angiogenic responses, and dendritic cells that are immunohistochemically closely linked to the mononuclear phagocyte system [3]. In both phases of IH growth, an increased number of mast cells have been found along the vessels, which show the expression of fibroblast growth factor (FGF) and are capable of the rapid but gradual release of vascular endothelial growth factor (VEGF). Therefore, mast cells are thought to play an important role that involves the stimulation of angiogenesis in the proliferation phase and the inhibition of angiogenesis in the involution phase, but their exact function in the growth of hemangioma is not yet fully understood [4,5,6]. Immunohistochemical study findings support the fact that hypoxia and hypoxia-inducible factor (HIF-2a) pathway activation, and the consequent overexpression of VEGF in endothelial cells, are involved in the pathogenesis of IH [7]. Few publications report on VEGF serum values in children with hemangiomas [8,9,10].

VEGF is an essential growth factor for vascular endothelial cells. It was previously known as a vascular permeable factor (VPF). It is a potent mediator of angiogenesis and vasculogenesis in fetuses, children, and adults [11,12,13]. During embryogenesis, VEGF regulates endothelial cell proliferation, migration, and survival, thereby regulating blood vessel density and size. After birth, VEGF maintains endothelial cell integrity and is a potent mitogen for microvascular and macrovascular endothelial cells. In adults, VEGF is involved mainly in wound healing and the reproductive cycle of women [13]. VEGF promotes vascular permeability in disease-affected tissues and is thought to contribute to tumor metastasis by promoting extravasation and angiogenesis [14]. Circulating VEGF levels correlate with disease activity in autoimmune diseases such as rheumatoid arthritis, multiple sclerosis, and systemic lupus erythematosus [15]. The potential role of VEGF in the pathophysiology of hemangiomas is thought to be probable. So far, there has been no clear conclusion about the usefulness of determining this proangiogenic factor in clinical practice, monitoring, and deciding on the treatment of children with IH [8,9,10].

The primary objective was to investigate the significance of determining VEGF serum levels at different stages of hemangioma growth in children. The secondary objectives were to determine VEGF serum levels in the proliferation and involution phases of IH, to compare VEGF serum levels in the proliferation and involution phases of IH with VEGF serum levels in a healthy control group of children comparable in terms of age and sex, and to determine the correlation of VEGF serum levels in children with IH with other clinical indicators (child’s age and sex, course of pregnancy, invasive prenatal procedures, prematurity, birth weight, clinical findings of solitary or multiple hemangiomas, pattern of hemangioma, type of hemangioma, extracutaneous manifestations, complications, associations with other anomalies in syndromes). The main hypothesis of the research was that in the proliferation phase, the VEGF serum levels are higher compared to the involution phase and the healthy control group.

## 2. Materials and Methods

For the purpose of the researched target group, samples were used of fifty (N = 50) children with IH without associated diseases at different stages of hemangioma growth (proliferative and involution phase), who were monitored and treated at the Children’s Hospital Zagreb during a three-year period (1 January 2013–31 December 2015). The children’s Hospital Zagreb is the largest tertiary center in the Republic of Croatia, where children from all over the country are referred for the treatment of infantile hemangiomas. The age range was from 0 to 18 years, and both sexes were included. The diagnosis of hemangioma was established via clinical examination. The control group consisted of one hundred (N = 100) healthy children (50 for the 1st phase and 50 for the 2nd phase), in whom the existence of IH and vascular malformations was ruled out via clinical examination. The subgroups of the control group (first and second phase of 50 children each) were chosen in relation to the proliferative and involutional phases of the target group. The groups were constructed from the patient cohort in such a way as to be comparable in terms of age and sex. This study was a nested case–control study. Informed consent for participation in the research was given by the parents. No patients were lost during the study. No biopsy was performed in children with IH. Blood sampling was performed routinely as part of the examination of the child, which was carried out at the Children’s Hospital Zagreb, during which there was an indication for blood sampling, so for the purpose of this study, no additional needle punctures were needed. For the purposes of determining vascular endothelial growth factor (VEGF), 5 mL of blood was taken from the patients on 2 occasions, at the time of the first examination in the hospital, when all anamnestic and clinical indicators of children with IH whose parents gave their consent to participate in the research were evaluated in detail, and, as a rule, after one to two years at one of the follow-ups when we could claim that the hemangioma was in the involution phase. A total of 5 mL of blood was taken from the controls on one occasion from children who routinely had blood drawn for some other reason, and for whom detailed anamnestic and clinical indicators determined that they did not have or had never had IH or vascular malformation (venous malformation, arteriovenous malformation, lymphatic malformation) and did not suffer from a tumor disease. Tubes with gel separators were used for serum, and the sample was allowed to coagulate for 30 min. Centrifugation was performed at 1000× *g* for 15 min. After the serum was separated, it was frozen at −20 °C. A Human VEGF Quantikine ELISA Kit (Catalog# DVE00, R&D Systems^®^, Minneapolis, MN, USA) was used to determine VEGF serum levels. A Quantikine Immunoassay Control Group (Catalog# QC01-1, R&D Systems^®^, Minneapolis, MN, USA) was used for quantitative control. The study was approved by the Ethics Committee of the Children’s Hospital Zagreb, School of Medine, University of Zagreb, Croatia.

### Statistical Analysis

Categorical variables are presented in the form of percentages and absolute frequencies. Differences were analyzed using Fisher’s exact test. The Wilcoxon test analyzed the dependent continuous variables, and McNemar’s test was used for categorical values. Continuous data are presented as medians (interquartile ranges) and arithmetic means (standard deviations). Differences in quantitative values between the test and control groups were analyzed using the Mann–Whitney *U* test and are presented as box-and-whisker plots. Multivariate regression analysis was performed to determine the relationship of VEGF serum levels with other clinical indicators. The analysis was performed using the software package R^®^ ver. 4.3.1 (R Foundation for Statistical Computing, Vienna, Austria), with the level of statistical significance set at *p* < 0.05.

## 3. Results

The group of children with infantile hemangiomas consisted of 36 (72%) female patients and 14 (28%) male patients (ratio 2.6:1). The average age of children in the proliferation phase of IH was 4.43 ± 2.43 months, while the average age of children in the control group for the first phase was 4.58 ± 2.51 months (*p* = 0.773). The average age of children in the involution phase was 23.58 ± 9.21 months, while the average age of children in the control group for the second phase was 23.54 ± 9.37 months (*p* = 0.904). The mean birth weight was 3258.06 ± 692.80 g. The mean treatment duration was 19.11 ± 10.82 months. The clinical characteristics of the patients with infantile hemangioma are shown in Table 1.

**Figure 1:**
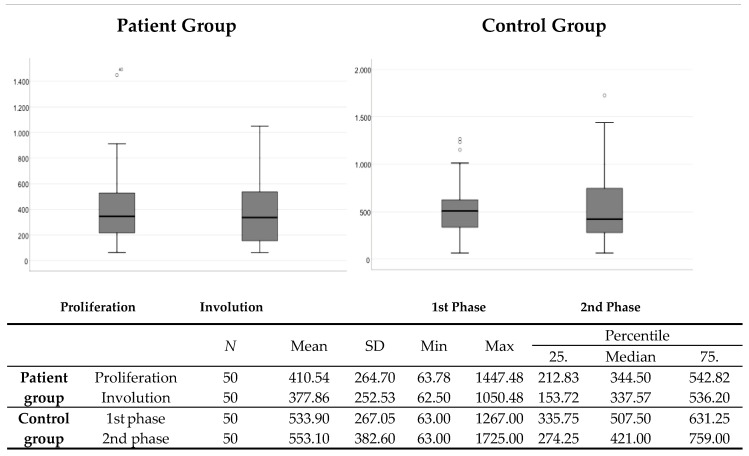
Comparison of serum VEGF levels between the proliferation and involution phases in the patient group and the phases of the control group comparable in terms of gender and age. N—number; SD—standard deviation; Min—minimum; Max—maximum.

There was no statistically significant difference in the comparison of serum VEGF levels between the proliferation and involution phases in the group of patients with IF (*p* = 0.171), or in the control group, which, for the purposes of this study, was divided into two groups (*p* = 0.981) (Figure 1).

**Figure 2:**
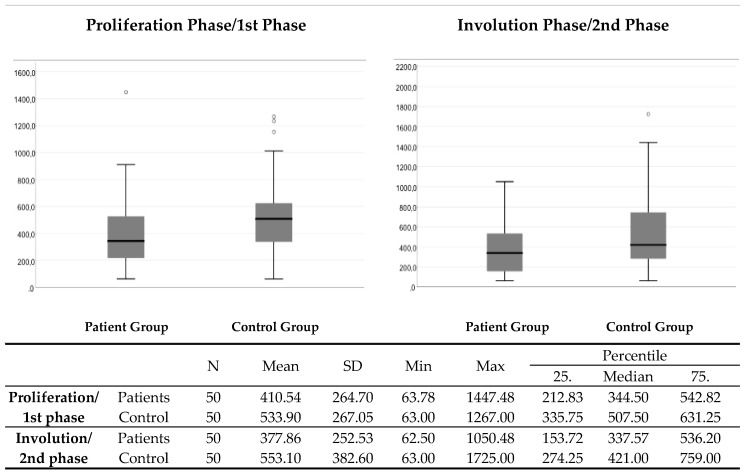
The comparison of VEGF serum levels between the patient group and the control group in the proliferation/1st and in the involution/2nd phase. N—number; SD—standard deviation; Min—minimum; Max—maximum.

The control group had significantly higher serum VEGF levels compared to the patient group in the proliferation/first (*p* = 0.009) and in the involution/second (*p* = 0.019) phase (Figure 2).

In the proliferation phase, the multivariate regression model explains 15% of the variance in the dependent variable, and there are no significant predictor variables (although complications were marginally positively related, *p* = 0.064). In the involution phase, the model explains 21% of the variance of the dependent variable, and the history of invasive prenatal procedures stands out as a significant predictor variable positively related to VEGF serum levels during the involution phase (beta coefficient = 0.33; *p* = 0.043) (Table 2).

## 4. Discussion

Although it is considered that a role of VEGF in the pathophysiology of hemangioma is likely [7], this study did not show statistically significantly higher values in serum VEGF levels in favor of the group of children with IH, compared to the control group, but on the contrary, we recorded statistically significantly higher values in the healthy group. Also, no statistically significant differences were found between the proliferative and involution phases in children with IH, which means that we cannot confirm our initial hypothesis in which we expected that serum VEGF levels would be higher in the proliferation phase compared to the involution phase, as well as that serum VEGF levels would be higher in the group of children with IH compared to the control group comparable in terms of age and sex.

Some studies have shown the importance of determining the serum level of VEGF by comparing the values in patients with IH in relation to patients with vascular malformations. It was concluded that it is useful to determine this in order to distinguish IH from vascular malformations [8,9,16,17]. In contrast to our results, the results of the study by Zhang et al. determined the existence of a statistically significant difference in serum VEGF levels between the proliferative and involution phases in favor of the proliferation phase, as well as a difference compared to the control group and the group with vascular malformations, leading to the conclusion that VEGF could be a specific marker of angiogenic activity and hemangioma growth [8]. In our research, one of the exclusion criteria was the existence of vascular malformations, so in this context, we were not able to obtain our own results and make observations.

A study by El-Raggal et al. also investigated the role of determining serum VEGF levels in differentiating IH from vascular malformations and their impact on the clinical course of IH. The study showed that there was no statistically significant difference in serum levels of VEGF in the phases of proliferation and involution in children with IH (*p* > 0.05), which is in accordance with the results of our study. The authors, analogously to our results, concluded that it is not useful to determine VEGF levels for the purpose of differentiating the growth phase of IH (proliferation or involution) [18]. Nevertheless, most studies support a strong association between increased tissue expression of VEGF in the phase of proliferation versus involution, and confirm the key role of VEGF in the regulation of angiogenesis in IH [19,20]. VEGF shows excessive tissue expression in IH, and its expression remains elevated during the early involution phase, which does not necessarily correlate with serum VEGF levels [21]. In relation to our study, according to a study by Przewratil et al., serum VEGF levels were statistically significantly higher in children with IH in the proliferation phase compared to the involution phase, compared to patients with vascular malformations and compared to a control group [9].

In our research, upon comparing VEGF between the two phases in the control group, no statistically significant differences were found, which points to the conclusion that it is not about variations in concentrations depending on the age of the children.

In a study by Rajewska et al., serum VEGF and serum placental growth factor (PIGF) levels were determined. The measurement was performed in two phases in children with IH, and a statistically significant difference was found in serum VEGF levels, which were higher in the proliferation phase (*p* = 0.02). It is significant that in ten patients’ serum, VEGF values were higher in the second measurement after 14 months, calling into question the usefulness of VEGF determination as a predictor of the hemangioma growth phase. No statistically significant difference was found in serum VEGF levels in children with IH compared to healthy controls. There was no statistically significant difference in PIGF values measured in the two phases [22].

Risk factors for the onset of IH from other publications are low birth weight (incidence of up to 30% in infants weighing less than 1.5 kg), advanced maternal age, female sex, white race, prematurity (25–29 weeks), multiple gestations, placenta previa, preeclampsia, hypoxia, and a positive family history of IH [23,24,25,26]. To analyze the relationship of VEGF serum levels in the proliferation and involution phases in children with IH with other clinical indicators, we used a multivariate linear regression model. There were no significant predictor variables in VEGF serum levels in children with IH in the proliferation phase with other clinical indicators (although complications were marginally positively related, *p* = 0.064). On the other hand, a significant predictor variable positively related to VEGF serum levels in children with IH in the involution phase was a history of invasive prenatal diagnostic testing (beta coefficient = 0.33; *p* = 0.043). In relation to the previously mentioned risk factors for the occurrence of IH in other studies [23,24,25,26], there is no doubt that they can be or are closely related to invasive prenatal procedures. In studies by Kaplan et al., Burton et al., and Bauland et al., an increased frequency of hemangiomas was observed in children whose mothers underwent transcervical chorionic villus biopsy (CVS), compared to mothers who underwent amniocentesis [27,28,29].

Various results have been reported in the literature regarding VEGF serum values in children with IH receiving oral propranolol therapy. In a study by Chen et al., in 22 patients with IH treated with propranolol per os, the average VEGF serum values after one month of treatment were 21.6% lower (*p* = 0.003), and they were 18% lower after three months (*p* = 0.63) with a significant correlation between lesion volume and VEGF values [30]. A study by Wu et al. showed that serum concentrations of VEGF, bFGF, and MMP-9 eight weeks after the administration of propranolol per os were significantly lower than before the treatment [31]. Moreover, a study by Makkeyah et al. showed that propranolol therapy induced a significant decline in VEGF levels after three months (*p* = 0.007) [32]. In contrast to these results, a study by Przewratil et al. did not confirm a significant decrease in VEGF in 50 children with IH after three months of propranolol therapy (*p* = 0.128), which is consistent with our study [33].

Although this is a non-invasive and objective method of measurement, according to the results of this study, the determination of VEGF serum levels using the currently available laboratory standards did not show the expected value and significance for clinical practice needs. The results of other studies are different, without a clear and definitive conclusion, and they often contradict each other, as is the case with the present study, in which the initial hypothesis states that there will be increased activity of VEGF in patients with IH. Therefore, further research will be needed to determine the definitive role of VEGF in the pathogenesis of IH.

### Limitations of this Study

The main limitation of this study is the small sample of patients with IH, so even if there are differences in the measured values, they hardly reach statistical significance. In order to monitor the movement of the same parameters in the natural course of hemangioma, this research, for ethical reasons, did not include patients who would only be “actively monitored” without treatment. Standardized regression coefficients were not calculated for the control group, and they could certainly bring certain insight regarding the relationship with the target group and higher VEGF serum levels. In this study, patients with vascular malformations were excluded; therefore, we could not compare the results with previous studies that drew conclusions based on the values of the observed parameters in differentiating IH from vascular malformations.

## 5. Conclusions

In our study, the hypothesis that children in the proliferation phase of hemangioma growth will have higher VEGF serum values compared to those in the involution phase was not confirmed. According to the results of this study, measuring VEGF serum levels has no significance in differentiating and determining the stage of growth of infant hemangiomas, and does not have the same value as the clinical examination and monitoring of patients. The VEGF values in the proliferation and involution phases in children with IH were unexpectedly lower compared to the results for children in the control group comparable in terms of age and sex. Therefore, measuring VEGF serum levels is irrelevant for clinical monitoring and deciding on the timing and the need for a therapeutic procedure in children with IH, i.e., it is incorrect to use serum VEGF levels as a basis for making treatment decisions for IH. Clinical examination and monitoring of children with hemangiomas remain the gold standard for the diagnosis, active monitoring, and treatment of children with IH.

## Figures and Tables

**Table 1:** Clinical characteristics of a group of patients with infantile hemangioma.

		*N*	%
Family history of IH	No	33	66
	Yes	17	34
Invasive prenatal procedures	No	42	84
	Yes	8	16
Prematurity	No	40	80
	Yes	10	20
Type of hemangioma	Superficial	2	4
	Deep	29	58
	Mixed	19	38
Localization of hemangioma	Segmental-face	20	40
	Focal	20	40
	Multifocal	10	20
Extracutaneous manifestations	No	45	90
	Yes	5	10
Complications	No	28	56
	Yes	22	44
Syndromes	None	48	96
	PHACE	1	2
	SACRAL	1	2
Indication for treatment	Vital	16	32
	Aesthetic/cosmetic	34	68
Treatment	Propranolol	18	36
	Propranolol with other medications	32	64
Side effects	No	43	86
	Yes	7	14
Relapse	No	47	94
	Yes	3	6
Treatment outcome	Complete resolution	37	74
	Proliferation	0	0
	No change in size	1	2
	Involution + residual lesion	12	24

N—number; %—percentage; IH—infantile hemangioma; PHACE—posterior fossa brain malformations, hemangioma, arterial lesions, cardiac abnormalities, and eye abnormalities; SACRAL—spinal dysraphism, anogenital, cutaneous, renal, and urologic anomalies, associated with an angioma of lumbosacral localization; SD—standard deviation.

**Table 2:** Multivariate regression model determining the relationship between the VEGF serum levels in children with hemangiomas in the proliferation and in the involution phase with other clinical indicators.

	Proliferation					Involution				
	Standardized Beta Coefficient	*t*	95% CI		*p*	Standardized Beta Coefficient	*t*	95% CI		*p*
			Lower	Higher				Lower	Higher	
Age (months)	−0.03	−0.14	−42.95	37.25	0.886	−0.08	−0.47	−45.32	28.36	0.644
Gender	−0.24	−1.39	−337.65	62.75	0.173	−0.17	−1.03	−277.15	90.67	0.311
Invasive prenatal procedures	0.02	0.10	−222.21	244.55	0.923	0.33	2.10	7.69	436.67	0.043
Prematurity	−0.34	−1.39	−540.73	100.83	0.173	−0.31	−1.31	−485.88	103.48	0.197
Birth weight (grams)	−0.15	−0.68	−0.23	0.11	0.502	−0.09	−0.42	−0.19	0.12	0.675
Type of hemangioma	−0.08	−0.48	−204.26	125.90	0.634	0.09	0.52	−112.61	190.68	0.605
Pattern of hemangioma	0.03	0.19	−104.22	125.45	0.853	0.07	0.44	−82.37	128.61	0.660
Extracutaneous manifestations	−0.13	−0.59	−503.35	276.19	0.559	0.06	0.31	−304.03	412.08	0.762
Complications	0.34	1.90	−11.38	373.22	0.064	0.21	1.21	−70.86	282.45	0.233
Syndromes	−0.11	−0.52	−697.68	412.30	0.606	−0.14	−0.69	−684.15	335.52	0.493
Treatment	−0.06	−0.33	−244.53	175.56	0.741	−0.16	−0.86	−274.94	110.97	0.395

*t*—*t*-value; *p*—*p*-value; CI—confidence interval.

## Data Availability

The data supporting this study’s findings are available upon request from the corresponding author.
